# Teicoplanin-Induced Acute Thrombocytopenia in a Patient With Foot Osteomyelitis

**DOI:** 10.7759/cureus.70341

**Published:** 2024-09-27

**Authors:** Charisma Marion Thomas, Husam Jamil

**Affiliations:** 1 General Medicine, Mid Yorkshire Teaching NHS (National Health Service) Trust, Wakefield, GBR; 2 Acute Internal Medicine, Mid Yorkshire Teaching NHS (National Health Service) Trust, Wakefield, GBR

**Keywords:** diabetes, drug-induced thrombocytopenia, glycopeptides, osteomyelitis, teicoplanin

## Abstract

This clinical case report explores the occurrence of acute thrombocytopenia following teicoplanin infusion in a patient with diabetic foot osteomyelitis. The patient, a 54-year-old male with a medical history of chronic kidney disease, hypertension, and type II diabetes mellitus, experienced a severe drop in platelet count during teicoplanin treatment. The article discusses the clinical presentation, diagnostic assessments, and management of teicoplanin-induced thrombocytopenia. Additionally, it delves into the broader context of drug-induced thrombocytopenia and emphasizes the importance of a systematic approach to understanding and managing this adverse reaction.

## Introduction

Drug-induced thrombocytopenia (DITP) is associated with a drop in platelet levels around a week of exposure to the medication and increased hemorrhagic risk with incidence rates of major and fatal bleeding of 9% and 8%, respectively [1]. Although, it is difficult to confirm thrombocytopenia is caused solely by a certain drug a paper by Bakchoul and Marini discusses several standards by which DITP can be diagnosed [1]. These are if thrombocytopenia occurred following exposure to the investigational drug, discontinuing the drug led to a significant recovery in platelet level and other potential causes of thrombocytopenia were eliminated, including medications that can lead to low platelet levels, either by reintroducing or continuing them during this period [1]. 

Teicoplanin, a lipoglycopeptide obtained from *Actinoplanes teicomyceticus*, which belongs to the Micromonosporaceae family, has attracted considerable interest due to its antimicrobial effects. It is particularly effective against gram-positive bacteria, as it binds to the D-alanyl D-alanine sequence, disrupting peptidoglycan synthesis and preventing the formation of the bacterial cell wall [2]. Teicoplanin is given intravenously via infusion or rapid injection because of its long half-life. It has been extensively used in European countries and the United Kingdom, although it has not received approval in the United States [2]. Teicoplanin is effective in treating deep-seated infections especially caused by *Staphylococcus* such as bacteremia, endocarditis, discitis, bone and joint infections namely osteomyelitis and septic arthritis. Teicoplanin, in comparison to other members of the same class, chiefly vancomycin, has a longer half-life and lower nephrotoxicity [3]. While teicoplanin presents a promising therapeutic option, its utilization is not devoid of complications. Hypersensitivity reactions, such as itching, fever, and pain, constitute common occurrences, while less frequent side effects include bronchospasm, headaches, leukopenia, ototoxicity, and thrombocytopenia. Rare adverse reactions, including abscess formation, agranulocytosis, angioedema, and neutropenia, have also been documented.

This article aims to elucidate a specific adverse reaction associated with teicoplanin, namely acute thrombocytopenia. Characterized by a rapid reduction in platelet count, thrombocytopenia poses a formidable challenge in the clinical management of affected patients. Through a detailed examination of this adverse event, the article seeks to contribute valuable insights into the complexities associated with teicoplanin therapy and its potential implications for patient care.

## Case presentation

**Figure 1:**
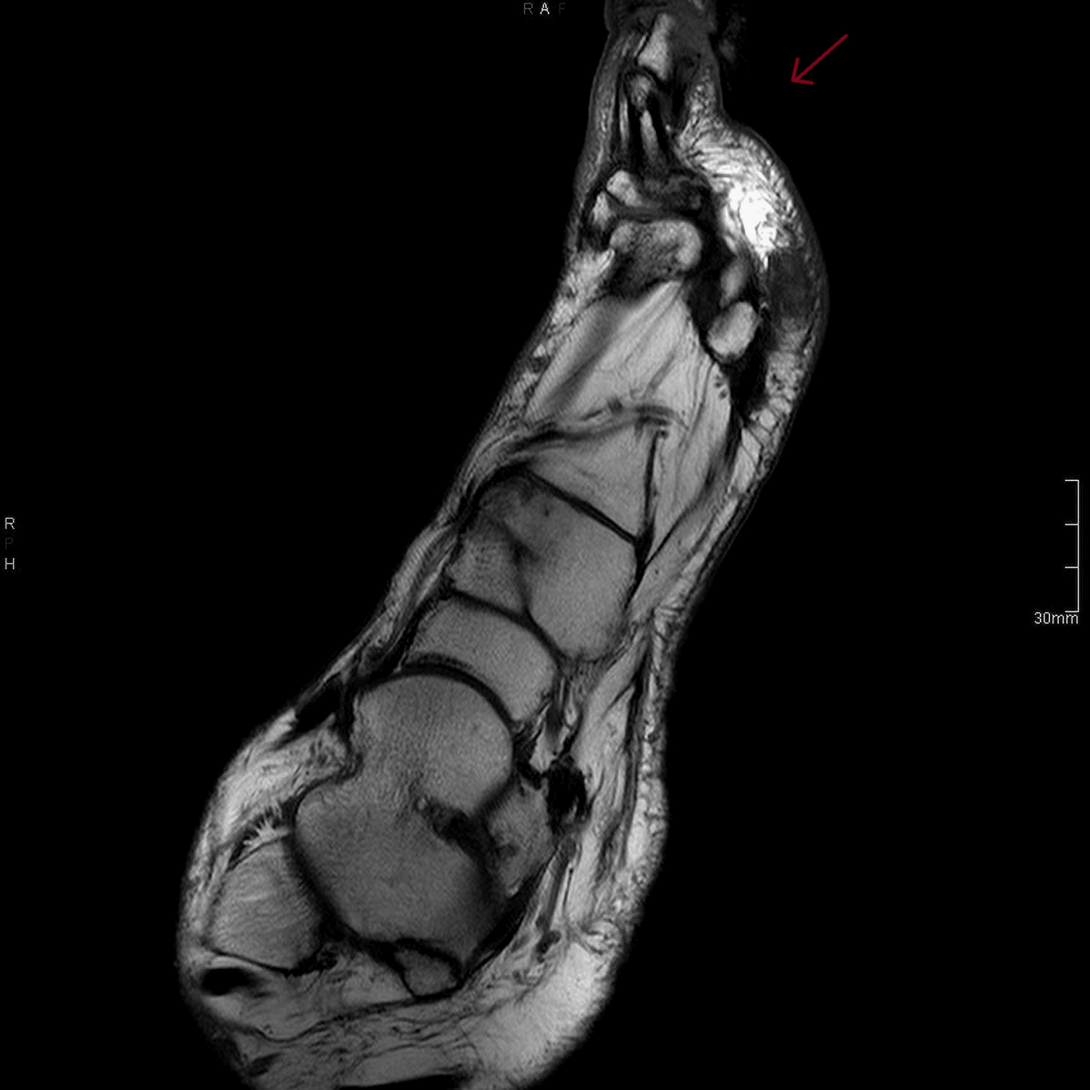
MRI foot right. MRI foot right reveals the destruction of the distal phalanx in keeping with osteomyelitis (red arrow).

We present a case of acute thrombocytopenia following teicoplanin infusion in a patient with diabetic foot osteomyelitis (Figure 1). The case involves a 54-year-old, Caucasian, British male with a medical history significant for chronic kidney disease (CKD) 3a and albuminuria category A2 (CKD), hypertension, and type II diabetes mellitus. Patient’s regular medication included anti-diabetic medication (Toujeo), anti-hypertensive drugs (amlodipine, atorvastatin), analgesics (fentanyl, oromorph, duloxetine), and anti-depressant (mirtazapine). No new medications were introduced other than teicoplanin during the admission. He was not known to have any drug allergies.

On admission, he presented with right foot pain. Teicoplanin was initiated according to guidelines because of a history of MRSA (methicillin-resistant *Staphylococcus aureus*). The patient's inflammatory markers consistently remained within the normal range. Tissue culture and sensitivity tests showed no abnormalities other than enrichment culture which grew *Staphylococcus epidermidis*, likely a contaminant. Hence, no adjustments were made to the antibiotic regimen. A pre-trough level should have been measured before the fifth day as per the guidelines; however, it was taken over seven days later, resulting in a level of 68.20 mg/L, which is considered acceptable from a therapeutic perspective.

On day 11 of teicoplanin, the patient’s platelet count plummeted to 11 x 10^9^/L. After consulting Hematology, the repeated platelet level reached a nadir of 2 x 10^9^/L (Table 1). The patient, however, was independent and mobile and had no active signs of bleeding. Two units of platelets were administered and on the following day the full blood count was repeated which showed a platelet level of 27 x 10^3^/L and it continued to gradually increase. In addition, teicoplanin was discontinued, and he was switched to oral co-trimoxazole to complete a total of six weeks antibiotic course.

**Table 1:** Full blood count. Table 1 depicts the different components of a full blood count panel with platelets 2 x 10^9^/L on day 11 of treatment with teicoplanin for diabetic foot osteomyelitis with normal hemoglobin levels indicating no active bleeding at the time when the patient was presenting with acute thrombocytopenia. Hb, hemoglobin; MCV, mean corpuscular volume; HCT, hematocrit; MCH, mean corpuscular hemoglobin; MCHC, mean corpuscular hemoglobin concentration; RDW, red cell distribution width.

Blood parameters	Results	Reference range
Hb (g/L)	136	130 - 180
WBC (x10^9^/L)	7.2	4.0 - 13.0
Platelets (x10^9^/L)	2	135 - 400
MCV (fL)	89.2	4.5 - 6.0
HCT (1/1)	0.396	80 - 98
RBC (x10^12^/L)	4.44	0.40 - 0.54
MCH (pg)	30.6	27 - 35
MCHC (g/L)	343	300 - 350
RDW (%)	13.0	0.00 - 14.0
Neutrophils (x10^9^/L)	5.09	2.0 - 7.5
Lymphocytes (x10^9^/L)	1.26	1.0 - 4.0
Monocytes (x10^9^/L)	0.60	0.2 - 0.8
Eosinophils (x10^9^/L)	0.22	0.04 - 0.4
Basophils (x10^9^/L)	0.07	0.0 - 0.1
Nucleated red cells (x10^9^/L)	0.0	0 - 0

Further blood tests were carried out as advised by Hematology such as coagulation screen D-dimer, lactate dehydrogenase (LDH), haptoglobin, blood film, hepatitis B virus (HBV), hepatitis C virus (HCV), HIV, antinuclear antibody (ANA), antineutrophil cytoplasmic antibodies (ANCA), rheumatoid factor (RF), immunoglobulin levels, serum electrophoresis, hematinics (Vit B12, folate, ferritin, iron). He was also examined for splenomegaly and chronic liver disease which excluded all other causes for thrombocytopenia. The patient showed a gradual increase in platelet level. A week later he was discharged with a follow-up at the diabetic foot clinic.

## Discussion

Drug-induced thrombocytopenia, a recognized adverse reaction linked to various medications, has been extensively studied. Six distinct pathologic mechanisms of DITP have been outlined by researchers such as hapten-dependent antibody reactions (e.g., penicillin), drug-dependent antibody reactions (e.g., quinine), fibrin-induced thrombocytopenia (e.g., eptifibatide), drug-specific antibody reactions (e.g., abciximab), autoantibody induction (e.g., L-dopa), and immune complex reactions (e.g., heparin) [4].

The pathogenesis of DITP involves two main mechanisms: decreased platelet production, as seen in chemotherapy, and accelerated platelet destruction. The latter is further categorized as non-immune mediated, as with antineoplastic agents, and immune-mediated [5]. A model proposed for drug-dependent antibody binding highlights the interaction between sensitizing drugs and platelets, particularly on sites like GP IIb-IIIa, GP Ib-V-IX, and platelet endothelial adhesion molecule-1 (PECAM-1). This model explains the reversible, noncovalent binding of drugs to platelets in a soluble form, facilitating a tight bond between antibodies and platelet epitomes. The subsequent production of drug-dependent antiplatelet antibodies occurs one to two weeks after drug exposure, elucidating how certain drugs may trigger an immune response leading to thrombocytopenia [6].

Forty-five percent of patients show the presence of Ig teicoplanin-dependent platelet reactive antibodies, primarily targeting the GPIIb/IIIa complex. Interestingly, the researchers detected these antibodies in 25% of the patients with normal platelet counts even though they were treated with teicoplanin. This implies that while the antibodies are associated with teicoplanin treatment, their presence does not necessarily lead to a reduction in platelet count in every patient. Thus, emphasizing the complexity of the mechanism involving teicoplanin-induced thrombocytopenia (TIT) [7]. It is relevant to note that there is low research on the incidence of TIT associated with clinical doses. In a recent cohort study of data collected from 482 patients utilizing the Naranjo scale, the incidence of teicoplanin-induced thrombocytopenia at a dosing range of 6-12 mg/kg/dose was reported to be 4.6% which is categorized as either possible or probable cases according to the Naranjo scale. This percentage suggests that out of 482 patients, only 22 experienced thrombocytopenia related to teicoplanin treatment. Additionally, the study highlights that none of the assessed patient variables were independently linked to an increased risk of thrombocytopenia, further supporting the conclusion that the occurrence of TIT is relatively uncommon, even among critically ill patients. Therefore, the findings suggest that while monitoring for adverse effects is important, the overall risk of developing TIT in this population is low [8].

Teicoplanin has demonstrated efficacy in outpatient treatment, particularly in addressing severe gram-positive infections, including complicated skin and soft-tissue infections, pneumonia, complicated urinary tract infections, endocarditis (streptococcal or enterococcal), and moderate to severe diabetic foot infections. Notably, its favorable pharmacokinetic profile, characterized by once-daily administration, has contributed to its use in diverse patient populations, with purported advantages over vancomycin, including a lower risk of nephrotoxicity [9].

In our clinical case, a comprehensive check was done regarding the medical history as well as patient’s drug history. On that note there have been studies on patients with CKD showing it to have contributed to mild thrombocytopenia, and only 8% were at risk of bleeding [10]. However, there is no evidence in our case accounting a relevant drop in platelet count since the patient was diagnosed with CKD. In terms of drug history, he was on antihypertensive and antidiabetic medications for years as mentioned previously without any evidence of acute thrombocytopenia on his blood results thus far. The only new change that the patient had undergone was the treatment with teicoplanin for his osteomyelitis. Almost two weeks into the treatment there was evidence of isolated severe thrombocytopenia. Although, the differentials were excluded through blood test and examinations recommended by Hematology. No additional tests were carried out to detect any antibodies (ELISA, Enzyme-Linked Immunosorbent Assay; IP-WB, Immunoprecipitation followed by Western Blotting). No routine blood test was carried out as the patient’s inflammatory markers (C-reactive protein, WBC) were consistently within normal range and no acute renal impairment since admission observe a trend/initial platelet drop.

As observed in our clinical case, the primary treatment for DITP is to discontinue the offending drug to prevent any further platelet destruction and for those with the severe bleeding risk can be treated with platelet transfusion to raise the platelet levels promptly. Post transfusion and ceasing the causative agent, the platelet level showed a gradual up trend.

## Conclusions

In conclusion, this clinical case report provides significant insights into the complexities of drug-induced thrombocytopenia, with a specific focus on teicoplanin. The report underscores the significance of routine investigations, even in the absence of overt clinical signs or symptoms of patient deterioration, as illustrated by the isolated case of severe thrombocytopenia following teicoplanin infusion. Moreover, the report advocates for ongoing research and clinical observation to foster a more comprehensive understanding of teicoplanin-induced thrombocytopenia in relation to clinical dose as well as its underlying mechanisms.

## References

[1] Bakchoul T, Marini I (2018). Drug-associated thrombocytopenia. Hematology Am Soc Hematol Educ Program.

[2] Zeng D, Debabov D, Hartsell TL (2016). Approved glycopeptide antibacterial drugs: mechanism of action and resistance. Cold Spring Harb Perspect Med.

[3] Schaison G, Graninger W, Bouza E (2000). Teicoplanin in the treatment of serious infection. J Chemother.

[4] Aster RH, Curtis BR, McFarland JG, Bougie DW (2009). Drug-induced immune thrombocytopenia: pathogenesis, diagnosis and management. J Thromb Haemost.

[5] Visentin GP, Liu CY (2007). Drug-induced thrombocytopenia. Hematol Oncol Clin North Am.

[6] George JN, Aster RH (2009). Drug-induced thrombocytopenia: pathogenesis, evaluation, and management. Hematology Am Soc Hematol Educ Program.

[7] Garner SF, Campbell K, Smith G (2005). Teicoplanin-dependent antibodies: detection and characterization. Br J Haematol.

[8] Elajez R, Abdallah I, Bakdach D (2022). Thrombocytopenia associated with teicoplanin use: a retrospective observational study. Ann Pharmacother.

[9] de Lalla F, Tramarin A (1995). A risk-benefit assessment of teicoplanin in the treatment of infections. Drug Saf.

[10] Dorgalaleh A, Mahmudi M, Tabibian S (2013). Anemia and thrombocytopenia in acute and chronic renal failure. Int J Hematol Oncol Stem Cell Res.

